# File-level malware detection using byte streams

**DOI:** 10.1038/s41598-023-36088-2

**Published:** 2023-06-01

**Authors:** Young-Seob Jeong, Medard Edmund Mswahili, Ah Reum Kang

**Affiliations:** 1 Department of Computer Engineering, Chungbuk National University, Cheongju, 28644 South Korea; 2 Department of Information Security, Pai Chai University, Daejeon, 35345 South Korea

**Keywords:** Mathematics and computing, Computer science

## Abstract

As more documents appear on the Internet, it becomes important to detect malware within the documents. Malware of non-executables might be more dangerous because people usually open them without worrying about inherent danger. Recently, deep learning models are used to analyze byte streams of the non-executables for malware detection. Although they have shown successful results, they are commonly designed for stream-level detection, but not for file-level detection. In this paper, we propose a new method that aggregates the stream-level results to get file-level results for malware detection. We demonstrate its effectiveness by experimental results with our annotated dataset, and show that it gives performance gain of 3.37–5.89% of F1 scores.

## Introduction

We are exposed to daily threat of malware programs. Non-executables (e.g., Microsoft word documents) might be more dangerous than executables (e.g., EXE files) because people open the non-executables without worrying about the inherent dangers; for example, we simply download and open attached documents when we get e-mails from colleagues or friends. As more non-executables are appearing on the Internet, it is becoming more important to detect the inherent dangers of malware.“

The malware detection is essentially a binary classification task on two classes (e.g., malware and normal). There have been studies for malware detection, and machine-learning (ML) models have shown quite successful results. However, using ML models have two limitations: (1) it requires much effort of domain experts for feature definition, and (2) it is poor at newly appeared malware because it takes time to find new features for dealing with the new malware. Deep-learning (DL) technique is a solution to the limitations, as it is known to extract arbitrary features from data without paying much human-effort on feature definition, and also known to be robust to newly appearing malware.

Recently, there have been few studies that applied the DL models to analyze byte streams for malware detection^1,2^. These studies are based on an assumption that the DL models better detect malicious actions within files by finding arbitrary patterns underlying the byte streams. They mainly exploited convolutional neural networks (CNN)^3^, that is known to be effective in capturing local patterns, and the CNN-based models achieved successful performance (e.g., accuracy, F1 score). These studies have the common limitation that their models are stream-level models; in other words, they are not designed for file-level malware detection, but for stream-level detection. They take a byte stream as input and predicts whether the given stream is malicious or not. However, a file may have one or more byte streams, and the file should be regarded as malware even if a single stream has malicious actions; this implies that the malware detection task is basically a file-level task. Such gap between the previous models and the task might cause low performance on the file-level detection.

In this paper, we propose a new method for malware detection using byte stream. Our method is designed to work in file-level by exploiting an aggregate function and the stream-level model. To the best of our knowledge, this is the first study that propose a file-level classifier for malware detection on non-executables. We conducted experiments with our manually annotated dataset collected from MS office documents (e.g., MS word, powerpoint, and etc.), and demonstrate that our method better detects the malware files than stream-level models.

## Preliminaries

### Malware detection on MS office files

There are two versions of MS office files: the 97-2003 version of the compound file binary format (.doc, .ppt, .xls) and the 2007 version of the OOXML structure (.docx, .pptx, .xlsx). In this paper, compound document files called Object Linking and Embedding (OLE) format were targeted. The OLE format has a structure similar to the File Allocation Table (FAT) file system manufactured by Microsoft. The concepts of files and folders in the file system are referred to as storage and streams in OLE format, respectively. An OLE file is largely divided into a header block and a data block. The header block has a size of 512 bytes and contains the main information of the entire OLE file. Magic ID exists in the range of 8 bytes from 0x0000 to 0x0007 in the header block, and it is usually a sequence of [D0, CF, 11, E0, A1, B1, 1A, E1] which is a signature keyword indicating that it is an OLE file. A data block is more than 512 bytes and has properties: stream data, Big Block Allocation Table (BBAT), and Small Block Allocation Table (SBAT). The properties hold information about files or folders in the device. BBAT is a link-type structure that includes stream location information inside OLE, and increases as the OLE file grows. SBAT stores a small area of data when entering a document. Stream data is the most important in an OLE file and takes up most of the data blocks. To extract byte streams from files, we can use python libraries such as olefile, zlib, BytesIO, and struct. The file header with olefile.OleFileIO and openstream functions are firstly extracted, and it is necessary to check file information such as properties, encryption, compression, and script inclusion. Once the list of streams in the OLE file is obtained, every stream is decompressed.

There have been studies of malware detection on MS office files. Yang et al.^4^ proposed a method for detecting MS-DOC malware using CNN models. Through static analysis, they found that the malicious MS-DOC files often have irregular file names and sensitive API calls. They showed that the malicious MS-DOC contains an encryption code to evade malware detection and a large number of meaningless characters. They converted MS-DOC files into 1024$\times$1024 gray images and processed them as input image. The results showed that the model had an average accuracy of 94.70%.

Mimura^5^ analyzed malicious MS office non-executable documents (e.g., .doc, .docx, .xls, .xlsx, .ppt, and .pptx) using language models. Malicious MS office documents were collected from VirusTotal and normal MS office documents were gathered from Stack overflow^6^. As most malicious MS office documents contain malicious VBA macros, this study checked if functions related to encoding, replacing, or splitting were included in VBA macro. Streams containing VBA macros are used to detect malware using language models and classifiers. Aishwarya et al.^7^ created MS office documents through Apache Poor Obfuscation Implementation (POI) and put some macro into randomly selected files. Apache POI allows to read or write MS Office file format in Java language, and supports Word, Excel, PowerPoint and the Open Office XML (OOXML) files (e.g., .docx, .xlsx, and .pptx). They analyzed the file structures of the complex file binary format (e.g., .doc, .ppt, and .xls) and OOXML format, and extracted features using oletools that is a Python package for malware analysis on MS Office documents. The feature set consists of more than 20 fields, and some features are based on fields such as Macro, AutoOpen, Suspicious, IOCs, HexStrings, Base64, Legitmate, Richtext, and DDElink. They exploited machine learning models such as random forest, Gradient boost and Ada boost algorithm for malware classification, and the random forest had the best accuracy of 96%. Most previous studies including above works mainly utilized a customized feature set obtainable from target files, but there are emerging recent studies that directly analyzes byte streams within files using deep learning techniques. In the next subsection, such studies are summarized and their common limitation is explained.

### Malware detection on byte streams

Malware detection on non-executables (e.g., MS Word file) is basically a classification task on two classes (e.g., normal and malware). There are two categories of approaches for the task: static and dynamic. The dynamic approach is to exploit a separated platform or an isolated virtual environment (e.g., virtual box), and analyzes step-by-step actions of a suspicious program. The existing studies of this approach have a common limitation that they are not reproducible as they usually exploit different emulations. On the other hand, the static approach is to directly analyze the target file without running it. Therefore, considering that we face more data everyday, the static approach will be preferable as it is relatively more efficient.

There have been studies of the static approach that uses handcrafted features and various machine-learning (ML) models such as support vector machines (SVM)^8^, logistic regression (LR), random forest (RF)^9^, and extreme gradient boosting (XGB)^10^. For example, Ranveer and Hiray^11^ trained the SVM with frequency-based patterns obtainable from executables, and it gave 95% of true positive rate (TPR). Morales-Molina et al.^12^ defined features on portable executable (PE) files and OpCode sequences, and achieved 89 and 96% of F1 scores using the RF model. Ajit Kumar et al.^13^ compared various ML models (e.g., decision tree, random forest, k-nearest neighbors, logistic regression, naive bayes, and linear discriminant analysis), and the best accuracy was 89.23%. Although the ML models have shown quite successful results, they have a common limitation that they require a huge effort of domain experts to find meaningful features.

**Figure 1:**
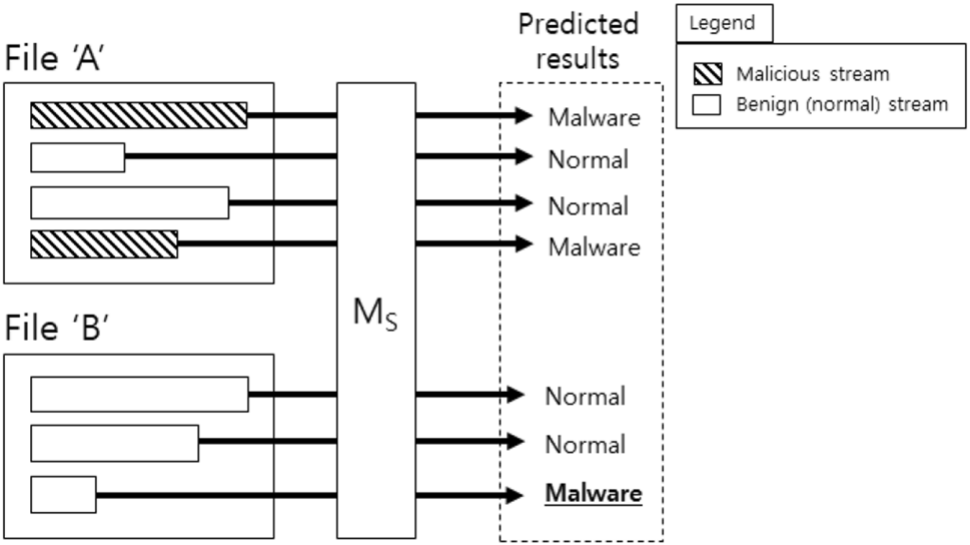
Stream-level malware detection, where $M_S$ indicates a stream-level model that predicts whether the given byte stream is malicious or normal.

Deep-learning (DL) technique is one of solutions for this limitation because it is able to learn underlying patterns or features automatically from given data. Especially, few studies exploited convolutional neural networks (CNN) for malware detection or classification, where CNN is known to effectively extract arbitrary local patterns. For example, Thosar et al.^14^ proposed a hybrid approach of gradient boosting and CNN for malware family classification, and achieved 93.53% of F1 score. Another studies applied the CNN models directly to the byte streams within non-executables. Raff et al.^1^ proposed a new shallow CNN architecture that takes as input a byte stream of PE header. Chen et al.^15^ formulated that the bytes in files are image pixels, and designed a CNN model for malware detection by capturing local patterns in the images. Jeong et al.^2^ used spatial pyramid pooling with average operation; it dramatically reduced the number of trainable parameters compared to other existing models without losing effectiveness (e.g., accuracy). In another paper of theirs^16^, they found that byte streams of different file formats (e.g., hangul word processor (HWP), portable document format (PDF)) allow CNN models to better learn underlying patterns for malware detection. These studies have shown potential of CNN models with byte streams, but they have a common drawback that their models work in stream-level. Figure 1 depicts how stream-level malware detection works. Each file contains arbitrary number of byte streams, and some streams may have malicious actions. Even if a file has only a single malicious stream, the file should be regarded as malware. With the stream-level model $M_S$ in Fig. 1, it misses only a single stream (i.e., the last stream) out of 7 streams, but it actually gave wrong prediction for the file ‘B’ out of two files; this implies that the model is poor for file-level prediction although it may seem fairly good for stream-level prediction. As far as we know, our paper is the first study of file-level malware detection for non-executables using byte streams.

## Method

Malware detection services are supposed to provide how likely a given file (or a set of files) contains malicious actions. Even if the file has only a single suspicious byte stream, the services must warn users about the danger. This implies that the services work in file-level, whereas previous studies using byte streams focused on developing stream-level prediction models (e.g., MalConv^1^, SPAP^2^). It is possible, of course, the stream-level models can be used for file-level prediction, as shown in Fig. 1; the model firstly generates per-stream predictions, and the file-level result will be ‘malware’ only if there is one or more ‘malware’ predictions in the per-stream results. However, the prediction model is designed to take as input a byte stream and generates a prediction, so it does not see file-level patterns (i.e., relation between byte streams). Such gap between the prediction model and services will probably lead to poor results (e.g., lower accuracy).

When we simply try to train the stream-level models to work in file-level, we face a challenging issue that the models must be applicable to varied number of streams. For example, in Fig. 1, the stream-level model $M_S$ takes as input four streams when it learns from file ‘A’, whereas it takes three streams for file ‘B’; but it is impossible to take such varied number of streams as input if we use conventional architectures such as fully-connected layers, convolutional layers, or pooling layers. Table 1 shows the statistics about the varied number of streams.
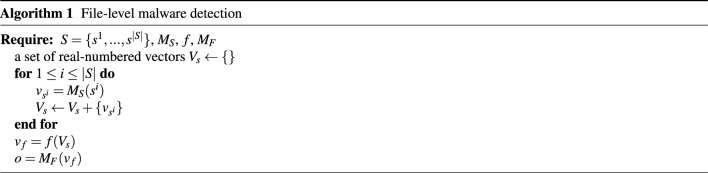

**Table 1:** The number of streams in a non-executable file.

	Mean	Variance
Malware	16.03	73.84
Normal	3.38	0.35
Total	9.65	76.76

**Figure 2:**
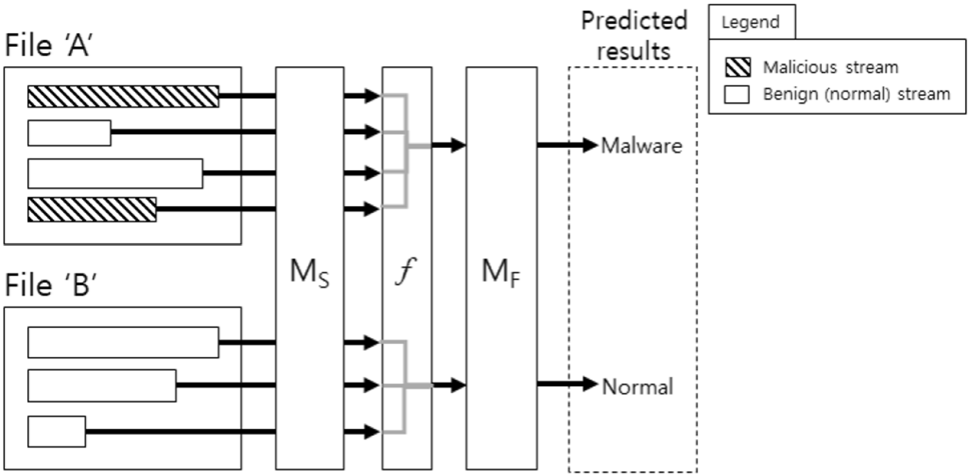
File-level malware detection, where $M_S$ indicates a stream-level model, $M_F$ is a file-level model, and *f* is an aggregate function.

As described in Fig. 2, our proposed method employs two models (i.e., $M_S$ and $M_F$) and the aggregate function *f*. More formal representation of our method is described via Eqs. (1), (2), and (3). Through the Eq. (1), given a set of padded streams $S = \{s^1, \ldots, s^i, \ldots, s^{|S|}\}$, we obtain real-numbered vectors $V_s = \{v_{s^1}, \ldots, v_{s^i}, \ldots, v_{s^{|S|}}\}$ where $|V_s| = |S|$, and $v_{s^i} \in {\mathbb {R}}^{m}$ is the output of $M_S$ for the i-th padded stream $s^i$. The Eq. (2) implies that the aggregate function *f* digests $V_s$ (i.e., set of arbitrary number of vectors) and gives a vector $v_f \in {\mathbb {R}}^{n}$. Finally, the output (i.e., two-dimensional vector or a probability of malware) is obtained from $M_F$ that takes the $v_f$ as input. Algorithm 1 provides a formal description of the steps.

1 $$\begin{aligned} v_{s^i}= & {} M_S(s^i) \end{aligned}$$

2 $$\begin{aligned} v_f= & {} f(V_s) \end{aligned}$$

3 $$\begin{aligned} o= & {} M_F(v_f) \end{aligned}$$

### Aggregate function

To deal with the varied number of streams, we defined an aggregate function *f* that digests outputs of $M_S$ into a single representation of *n*-dimensional real-number vector, as shown in Fig. 2. For example, if we deal with the file ‘A’, then the four streams with different lengths are firstly padded, so they become to have the same length. Here, we generalize the functionality of $M_S$ that takes each stream as input and gives *m*-dimensional vector as output. If $M_S$ is assumed to be a multi-layered network, then it will give vectors of different dimensions as we pick different layers; for example, when $M_S$ has three layers of 100, 50, and 2 dimensions, then *m* might be 100, 50, or 2 depending on which layer we determined to generate output of $M_S$. When we obtain four vectors of *m* dimensions from the four streams of file ‘A’, then they are passed into the aggregate function *f* that generates *n*-dimensional representation as output. While there are many options (e.g., hash function) as the aggregate function, we chose ‘average’ function that generates an averaged vector from the varied number of vectors; $m = n$ when we use the average function because the function generates an averaged value for each dimension.

### File-level classifier

 With the *n*-dimensional vector as a feature vector generated by the aggregate function, we train a file-level classifier $M_F$. The classifier might be any conventional models such as linear classification models, neural networks, or support vector machines. In this paper, we used a *k*-dimensional fully-connected layer followed by a two-dimensional output layer. The difference between $M_S$ and $M_F$ is that the file-level classifier $M_F$ is trained with a *n*-dimensional feature vector of every file, whereas the stream-level classifier $M_S$ is trained with a padded byte stream.

### Ethical standards

This study does not involve data of human participants or animals. We have no potential conflicts of interest.

## Experiments

### Data and settings

We collected malware and normal files of Microsoft (MS) office. Malicious files were obtained from a Korean anti-virus company, and normal files were collected from public portal site operated by the Korean Ministry of the Interior and Safety. We extracted all streams from malicious and normal files in bytes, and if the stream is compressed, it was extracted after decompressing it. The stream data is available online.

The data is split into three subsets: training, validation, and test set, as summarized in Table 2. The imbalance of streams is caused by the extremely high variance of the number of malware streams, as shown in Table 1. Streams belonging to the same file are placed in the same subset. We used a machine of Intel(R) Xeon(R) Silver 4214 CPU @ 2.20 GHz 48 cores and four graphics processing unit (GPU) of NVIDIA Quadro RTX 5000. All models are implemented using Python3 language with Tensorflow packages.

**Table 2:** Data statistics.

		Malware	Normal	Total
File	Train	413	432	845
	Validation	45	48	93
	Test	68	56	124
Stream	Train	6749	1455	8204
	Validation	688	158	846
	Test	996	199	1195

### Results

We employed two recent CNN models, MalConv^1^ and SPAP^2^, as $M_S$, and compared them by performance (e.g., accuracy). The convolutional layers of MalConv have 128 dimensions with kernel size of 500, while the consecutive convolutional layers of SPAP have 64 and 264 dimensions. We made them to have the same dimension of 128 at the layer just before their output layers, and took 128-dimensional real-number vectors from the layer as the output of $M_S$; in other words, $m = 128$ as we picked the layer just before their output layer. When we train the SPAP, batch normalization^17^ and drop-out techniques^18^ are used for convolutional layers and fully-connected layers, respectively. For the MalConv, the drop-out is applied to its fully-connected layers. The parameters of MalConv and SPAP models were updated for 20 and 15 epochs, respectively, using cross-entropy loss function and Adam optimizer^19^ with initial learning rate of 0.001, where the hyper-parameter setting is determined by a grid searching. We also employed a sample weight technique to mitigate the imbalance of the dataset; that is, the data instances are weighted using a ratio between two classes (i.e., malware and normal). Note that it is not our purpose to improve performance of these two stream-level models, but to prove our proposed method works better than just using the stream-level models for file-level malware detection.

**Figure 3:**
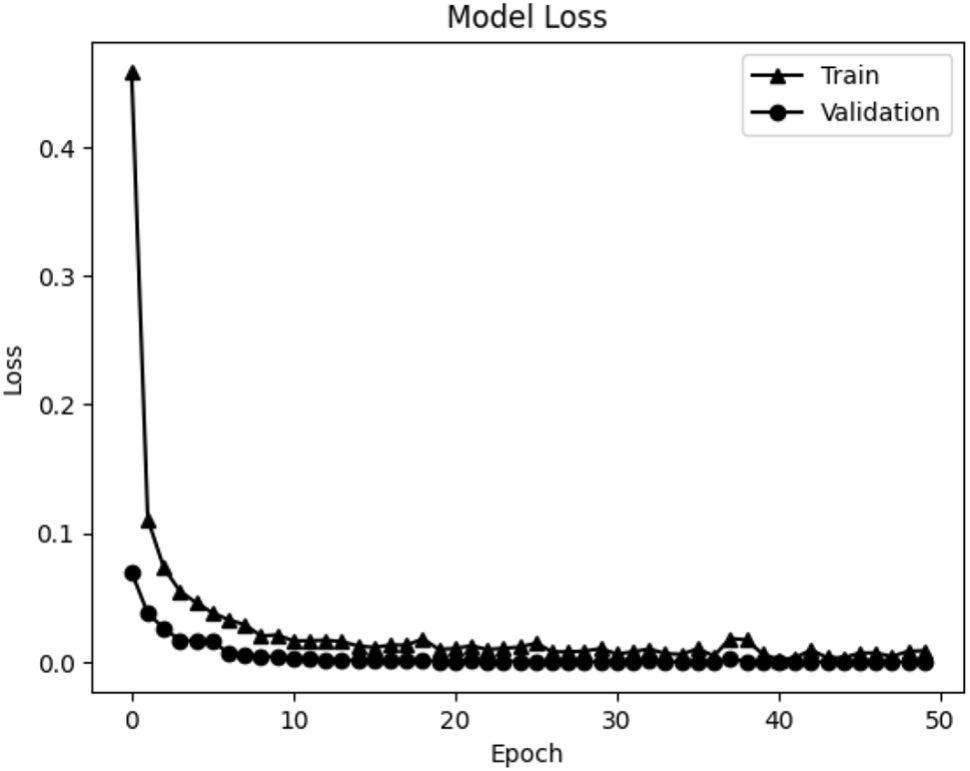
Loss plot of the file-level classifier, where the horizontal and vertical axis are loss and the number of epochs, respectively.

We used ‘average’ function as the aggregate function *f* that computes averaged value for each dimension of varied number of 128-dimensional vectors that came from the $M_S$. We set $k = 32$, so the file-level classifier $M_F$ has a 32-dimensional fully-connected layer with a drop-out followed by a 2-dimensional output layer with a softmax function. The parameters of $M_F$ were updated for 10 epochs using cross-entropy loss function and Adam optimizer with initial learning rate of 0.001. Figure 3 is a plot of training and validation loss of the file-level classifier. During the training phase of $M_S$, the parameters of $M_F$ were not updated.

Table 3 summarizes the results of file-level malware detection, where the precision, recall, and F1 scores are averages of three independent experiments. Our method exhibited a significant improvements compared to the stream-level models; for example, our method with SPAP achieved 91.12% of F1 score for malware class whereas using SPAP only gave 85.23% of F1 score. The results imply that our method generally gives 3–6% improvements of F1 scores. Such a big performance gap is due to the inconsistency between the task and the models. That is, the stream-level models are designed and trained for the task of ‘stream-level’ prediction, so they must be poor on the ‘file-level’ prediction. On the other hand, our method is designed for the file-level prediction by considering relational information between streams within a given file.

**Table 3:** Results of file-level malware detection, where $\triangle$ indicates how much performance was improved by our method.

	Precision (%)		Recall (%)		F1 score (%)	
	Malware	Normal	Malware	Normal	Malware	Normal
MalConv	97.46	76.28	75.00	97.62	84.77	85.64
SPAP	96.42	77.20	76.47	96.43	85.23	85.71
Ours (with MalConv)	96.61	82.67	83.33	96.43	89.47 ($\triangle$4.70)	89.01 ($\triangle$3.37)
Ours (with SPAP)	96.26	86.07	86.77	95.83	91.12 ($\triangle$5.89)	90.55 ($\triangle$4.84)

When we look at precision, the models commonly suffer from low performance in normal class, probably due to the class imbalance as shown in Table 2. our method gives significant improvements in the normal class, without losing much precision of malware class; this in turn allows our method to have greater F1 scores. A similar phenomenon appears in recall, and our method again gives improvements in malware class. We also observed that our method gives better performance when it works with SPAP. This implies that using better stream-level model as $M_S$ probably contributes to better file-level performance.

## Discussion

The two stream-level models, MalConv and SPAP, exhibited comparable results as shown in Table 3, while the SPAP is slightly better in terms of the F1 scores. Both models have shown better results when they work with our method. The reason of such performance gap is that our method eliminates the inconsistency between the stream-level models and the file-level task. Our aggregate function plays a crucial role in here, as it makes a fixed dimensional vector out of varied number of byte streams in each file.

Our method might be seen that we just use the stream-level models $M_S$ as feature generators and train a shallow neural network as a file-level classifier. It is worth noting that it is not trivial to use $M_S$ as feature generator because there are varied number of streams in a file; that is, the dimension of feature vector varies if we simply concatenate the output vectors of $M_S$, so it is not possible to use it as a feature vector for the file-level classifier. Therefore, the aggregate function *f* plays a crucial role in our method because it allows the feature vector to have a fixed dimension.

In this paper, we basically chose the ‘average’ function as the aggregate function *f*. We also investigated some other functions to check how important the aggregate function is. Table 4 summarizes the results with two different aggregate functions: ‘max’ and ‘min’. Interestingly, these two functions did not give much performance improvements compared to the results of stream-level models. This might be explained that the generated vectors by the stream-level models carry meaningful information of the varied number of streams, and ‘min’ or ‘max’ operation may lose such meaningful information as they keep only the smallest or greatest values.

**Table 4:** Results of file-level malware detection using different aggregate functions.

	Precision (%)		Recall (%)		F1 score (%)	
	Malware	Normal	Malware	Normal	Malware	Normal
Ours (with MalConv, *f* = max)	98.05	75.69	74.02	98.21	84.35	85.49
Ours (with MalConv, *f* = min)	97.58	79.25	78.92	97.62	87.25	87.47
Ours (with SPAP, *f* = max)	97.00	80.17	79.90	97.02	87.52	87.73
Ours (with SPAP, *f* = min)	92.32	78.63	79.41	91.97	85.38	84.78

## Conclusions

In this paper, we proposed a new method of file-level malware detection. The proposed method consists of a stream-level model $M_S$, an aggregate function *f*, and a file-level classifier $M_F$. By experimental results, we demonstrated that our method works better than previous stream-level models. We trained $M_S$ firstly, and used their output vectors to generate a fixed length of feature vector using *f*. As a future work, we plan to design a new file-level model that does not require to train such stream-level models. We are also looking for better aggregate functions instead of the ‘average’ function, and perform experiments with other stream-level models.

## Data Availability

The stream data used in this study is available for non-commercial use: https://sites.google.com/arkang.net/malwarebytestreams.
